# Governance and mental health: contributions for public policy approach

**DOI:** 10.1590/S1518-8787.2017051006991

**Published:** 2017-01-23

**Authors:** Lina Díaz-Castro, Armando Arredondo, Blanca Estela Pelcastre-Villafuerte, Marc Hufty

**Affiliations:** I Servicios de Atención Psiquiátrica. Secretaría de Salud. Ciudad de México, México; II Instituto Nacional de Salud Pública. Cuernavaca, Morelos, México; III Graduate Institute of International and Development Studies. Geneva, Switzerland

**Keywords:** Mental Health Services, Public Health Policy, Review, Policy Making

## Abstract

**OBJECTIVE:**

To analyze the conceptualization of the term governance on public mental health programs.

**METHODS:**

In this systematic review, we analyzed the scientific literature published in the international scenario during 15 years (from 2000 to 2015). The databases analyzed were: Medline, CINAHL, PsycINFO and PubMed. Governance and mental health were the descriptors. We included relevant articles according to our subject of study and levels of analysis: (i) the concept of governance in mental health; (ii) process and decision spaces; (iii) strategic and pertinent actors who operate in the functioning of the health system, and (iv) social regulations. We excluded letters to the editor, news articles, comments and case reports, incomplete articles and articles whose approach did not include the object of study of this review.

**RESULTS:**

We have found five conceptualizations of the term governance on mental health in the area of provision policies and service organization. The agents were both those who offer and those who receive the services: we identified several social norms.

**CONCLUSIONS:**

The concept of governance in mental health includes standards of quality and attention centered on the patient, and incorporates the consumers of mental healthcare in the decision-making process.

## RESUMEN

**OBJETIVO:**

Analizar la conceptualización del término gobernanza en las políticas de salud mental.

**MÉTODOS:**

En esta revisión sistemática se analizó literatura científica publicada en el ámbito internacional durante 15 años (de 2000 hasta 2015). Las bases de datos analizadas fueron: Medline, CINAHL, PsycINFO y PubMed. Los descriptores fueron gobernanza y salud mental. Fueron incluidos artículos relevantes de acuerdo a nuestro objeto de estudio y niveles de análisis: (i) concepto de gobernanza en salud mental; (ii) proceso y espacios de decisión; (iii) actores estratégicos y de interés que intervienen en el funcionamiento del sistema de salud, y (iv) normas sociales. Se excluyeron cartas al editor, noticias, comentarios y reporte de caso, artículos incompletos y artículos que no incluyeran en su abordaje el objeto de estudio de esta revisión.

**RESULTADOS:**

Se reportaron cinco conceptualizaciones del término gobernanza en salud mental en el ámbito de políticas de provisión y organización de servicios. Los actores fueron desde proveedores a usuarios de servicios; se identificaron diversas normas sociales.

**CONCLUSIONES:**

El concepto de gobernanza en salud mental incorpora estándares de calidad y atención centrada en el paciente, e incluye a los usuarios en la toma de decisiones.

## INTRODUCTION

There are different concepts of the term governance in the health systems^11^. We will focus on the part of the direction, defined as a group of established tasks and functions used to achieve control and responsibility in order to improve the system^19^ optimization, by offering quality services and protecting the right to health^38^.

A large number of researchers in international congresses have analyzed the role of governance in public policies in health. They mention the different definitions of governance^5^, a process to which different interests merge, many times conflictive^23^ ^,^ ^41^. If we see as indispensable and as basic condition the interaction between the academic world and those who take decision, it is essential that we review the keypoints to governance^33^.

There has been increasing world literature on this subject, but there is not a consensus on the definition of governance. Some international organizations equate the concept of governance with that of stewardship, management and governability, still bearing in mind important semantic differences^7^ ^,^ ^24^ ^,^ ^30^ ^,^ ^a^ ^,^ ^b^ ^,^ ^c^.

The World Health Organization’s definition of global governance^b^ ^,^ ^d^ refers to the implementation of policies and practices that promote equitable health systems. Other definitions, such as governance in health^4^, arose to refer to actions adopted by a society arranged to promote and protect health. We address the focus on management, notwithstanding its quite different scope. Intersectoral governance^18^ refers to the set of political, legal and organizational structures that enable the coordination of multiple sectors to address health problems, under a normative approach. These definitions either are different under the same approach or have a different and unclear approach, as expressed in the following definition: the administration is part of the governance process inasmuch as it refers to application policies and decisions, in order to achieve the intended results^20^ ^,^ ^28^. Another study conceptualizes governance as an analytical tool for understanding the factors in organizing the interaction of actors, the process dynamics and the game rules^c^ ^,^ ^e^. Most definitions refer to governance as a set of functions of the health system^9^ ^,^ ^16^. Within each country, the reach depends on the social context of the health system, the existing policies and the range of issues that are to be solved ^4^ ^,^ ^17^ ^-^ ^19^ ^,^ ^25^ ^,^ ^31^ ^,^ ^45^ ^,^ ^d^ ^,^ ^f^.

The governance in mental health is a relatively unexplored field, in contrast with the overall health system. The need for increasing the research on mental health systems gives rise to an interest in an approach that involves how the governance term is referred to.

The governance model in the field of mental health needed to have a limited, observable, reproducible and generalizable scope. We identify the study of governance through an analytical^e^ and methodological framing: the Analytical Framework of Governance^22^. It is a generalizable, non-normative concept that defines governance as a social fact, endowed with analyzable and interpretable characteristics, in the sense that every society develops its own ways of governance, its decision-making systems or conflict resolution among its members, norms and institutions^22^.

This analysis was conducted through the observable constituent elements of governance^22^: actors, social regulations, process and nodal points, in order to identify the extent of their influence over mental health policies in decision-making spaces^e^. These elements are defined as follows:

- Actors: Every individual, organization or group involved in managing any institutional aspect for the purpose of reaching agreements on addressing concrete problems on a collective plan^36^.
- Norms: System of values, principles and agreements for the successes and advances. The arrangements between the actors are shaped by several norms, both formal and informal. They guide the actors’ behavior and are modified by collective action. The formal norms may take different forms: meta-norms, principles that guide the social contract in a broader sense; constituent norms, that define the organization of the group or the sectoral institutional framework; regulatory norms, that define the conduct guidelines that establish appropriateness from a societal point of view, in terms of behavior, i.e., what every person should do^22^. The informal norms are based on belief systems and cultural values.
- Process: A number of different phases or stages through which a system go to achieve a goal, a product or a service. We identify sequences that help evaluate the direction in which such processes evolve and that help determine the factors related to change^22^.
- Nodal points: Spaces for interaction among actors that accommodate the confluence of social interactions defined in physical spaces, be they real or virtual, with the convergence of actors, processes and norms that produce isolated or interactive effects^22^.

The objective of this study was to analyze the conceptualization of the governance term in mental health policies.

## METHODS

Systematic review and analysis of the published literature on mental health governance at the international level for a period of 15 years, from the year 2000 to the year 2015.

The period extended from the year 2000, inasmuch as 2001 was declared “the Year of Mental Health”. Documents related to governance, specifically for mental health systems, were drafted and published during some months of that year.

The systematic review was reported based on the recommendations proposed in the PRISMA (*Preferred Reporting Items for Systematic Review and Meta-Analysis*) Declaration^f^ ^,^ ^g^.

We identified 21 articles about the subject of governance in mental health. The scientific literature search was conducted from April to June 2015 and took place in four databases: Medicine, CINAHL, PsycINFO and PubMed. Gray literature was not included.

Along preliminary review of several literature terms and MeSH term definitions in the databases, the keywords were chosen in order to identify relevant scientific articles in the research on governance on mental health. The descriptors were “governance” and “mental health”. We also examined the search terms in databases that include publications in Spanish language in the Virtual Health Library (VHL), such as Lilacs and SciELO. These databases were shown to contain only articles related to the governance term but unrelated to mental health. Consequently, they were not included in the review.

The search for the terms “governance” and “mental health” in any part of the text resulted in 4,708 articles. By limiting the search with the inclusion of “governance” in the title, 314 articles were identified. By defining the search parameters with both terms in the title of the article, the search resulted in 119 articles.

We reviewed the titles and the abstracts of the 119 articles, in terms of content, according to the following inclusion and exclusion criteria:

- Inclusion criteria. Relevant articles according to our subject of study and levels of analysis: (i) the concept of governance in mental health; (ii) process and decision spaces; (iii) strategic and pertinent actors who operate in the functioning of the health system, and (iv) social regulations.

According to their types, the articles included were: research articles, original articles, brief research articles, special section, review articles, case studies, author manuscripts, and newspaper articles.

- Exclusion criteria. Letters to the editor, news articles, comments and case reports, incomplete articles and articles whose approach did not include the object of study of this review.

Out of the 119 articles, 21 were duplicated; 11 were excluded for being documents such as letters to the editor, news articles and comments; one article was excluded because its year of publication did not correspond to those established; 51 articles addressed subjects that did not correspond to the object of study (10 from Medline, CINAHL and PsycINFO and 41 from PubMed).

We selected 35 articles in full text for comprehensive review. We applied a checklist to them according to a form adapted from the Scottish Intercollegiate Guidelines Network (SIGN), which evaluated the quality and evidence criteria, in accordance with the following: a) enough evidence to respond to the objective; b) studies consistent in their conclusions; c) studies relevant to our objective (similar subject of study); d) concern with the publication bias (origins of the studies, research teams, organizations); e) aimed benefits; f) viability, if the study applies to the context; g) recommendations, development based on evidence and future research.

**Figure 1:**
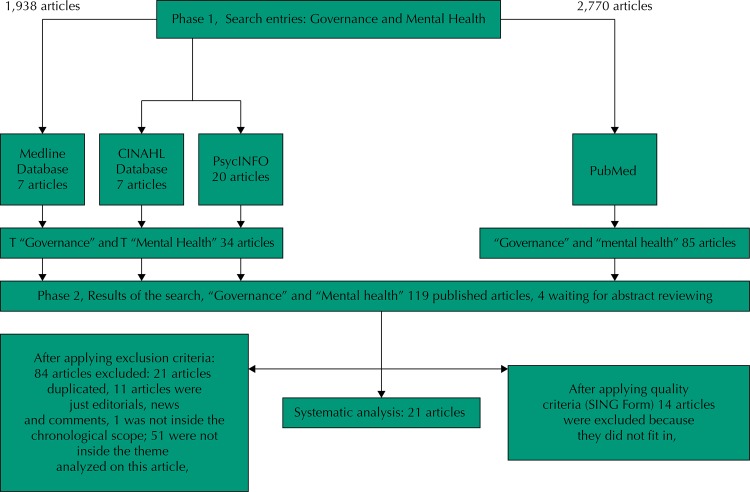
Diagram of the systematic data flow.

Fourteen articles were excluded for not meeting the proposed quality criteria. For the sake of systematic analysis 21 articles were included (Figure 1).

## RESULTS

Out of the 21 studies, most were published in the year 2010 (33.3%), carried out in the United States, United Kingdom, South Africa and other countries (Table 1). 33.0% referred to the concept of governance; almost all studies focused on actors of mental health (95.0%) and among them more than half referred to strategic actors. Likewise, a high percentage of articles referred to social regulations (71.0%), with a poor approach on the categories process and nodal points (Table 2).

**Table 1:** Classification of the articles of governance on mental health.

No.	Author	Year	Country/Region	Magazine	Level of analysis			

					Actor	Pattern	Nodal point	Process
1	Rogers et al.^37^	2002	ENG	J Ment Health	X			X
2	Wolff N^46^	2002	UK	J Health Polit Polic	X	X	X	
3	Newman et al.^32^	2003	UK	J Intell Disabil Res	X			X
4	Linhorst et al.^26^	2003	USA	Soc Serv Rev	X	X	X	X
5	Singh B^39^	2003	AU	J Ment Health	X	X	X	
6	Callaly et al.^8^	2005	AU, NZ	Australas Psychiatry	X			
7	Arya et al.^2^	2005	AU, NZ	BMC Health Serv Res	X			X
8	Meenaghan et al.^29^	2007	ENG	Journal of Mental Health	X	X		X
9	Gask et al.^15^	2008	ENG	BMC Health Serv Res	X	X		X
10	O´Connor et al.^35^	2008	AU	BMC Health Serv Res	X	X		X
11	Draper et al.^13^	2009	SA	Health Policy Plann	X	X	X	
12	Curtis et al.^10^	2010	USA	Psychiatr Rehabil J	X	X	X	X
13	Drake et al.^12^	2010	USA	Psychiatr Rehabil J	X	X	X	X
14	Lund et al.^27^	2010	SA	Soc Psychiat Epidemiol		X		
15	Niemi et al.^34^	2010	VN	BMC Health Serv Res	X	X		X
16	Stelk et al.^40^	2010	USA	Adm Policy Ment Health	X	X		
17	Van Houtte E.^43^	2010	USA	BMC Health Serv Res	X	X		
18	Woltmann et al.^47^	2010	USA	Psychiatr Rehabil J	X			
19	Yasami et al.^48^	2011	ET, IN, NP, SA, UG	PLoS Med	X			
20	Frueh et al.^14^	2012	USA	B Menninger Clin	X	X		X
21	Hodges et al.^21^	2013	USA	Admin Soc Work	X	X		X

**Table 2:** Constitutive elements of the governance in mental health.

Category of analysis	Number of articles	Percentage of articles
Concept of governance	7	33.3
Actor	20	95.0
Strategic	20	95.0
Interest	10	48.0
Social standards	15	71.0
Meta standards	10	48.0
Constitutive standards	8	38.0
Regulative standards	8	38.0
Informal standards	4	19.0
Nodal points	6	29.0
Physical space	4	19.0
Virtual space	5	24.0
Processes	12	57.0
Clinical scenarios	12	57.0
Other scenarios, i.e., non-profit work. Not consolidated	8	38.0

### Conceptualization of the Governance Term in Mental Health

In two studies there was the conceptualization of clinical governance as a framework under which the organization of the national system of mental health is responsible for the continuous improvement of the service quality^8^ ^,^ ^32^. In another study, the concept of governance referred to a research framework as a risk management process to the patients, users and other people under medical attention^29^. Another definition was shared governance, which concerns the decision-making process by experts, reflects values, processes focused on the patient and evidence-based medicine^10^ ^,^ ^12^. One more definition: collaborative governance, i.e., a process of interinstitutional collaboration in service delivery, structure that provides clarity as for the roles, responsibilities and accountability^21^. The definition of governance in mental health referred to all the agencies that act to govern national health services, and determine the service structures, the key actors in policy-making, service delivery and results in mental health^34^.

These five concepts of the governance term in mental health found^8^ ^,^ ^10^ ^,^ ^12^ ^,^ ^21^ ^,^ ^29^ ^,^ ^32^ ^,^ ^34^ refer to the clinical setting, the mental health services and results^8^ ^,^ ^10^ ^,^ ^34^. We noted that the governance process must be responsible, participatory and accountable (Table 3).

**Table 3:** Governance, focus on mental health policies.

Governance	Chronology of authors																						
Focus on mental health policies	Rogers et al.^37^ (2002)	Wolff N.^46^ (2002)	Newman et al.^32^ (2003)	Linhorst et al.^26^ (2003)	Singh B.^39^ (2003)	Callaly et al.^8^ (2005)		Arya et al.^2^ (2005)	Meenaghan et al.^29^ (2007)		Gask et al.^15^ (2008)	O´Connor et al.^35^ (2008)	Draper et al.^13^ (2009)	Curtis et al.^10^ (2010)	Drake et al.^12^ (2010)	Lund et al.^27^ (2010)	Niemi et al.^34^ (2010)	Stelk et al.^40^ (2010)	Van Houtte E.^43^ (2010)	Woltmann et al.^47^ (2010)	Yasami et al.^48^ (2011)	Frueh et al.^14^ (2012)	Hodges et al.^21^ (2013)
Framework in mental heath policies																							
Services supply	X	X					X	X			X*	X	X	X		X	X	X		X	X	X*	X
Organization of services			X	X	X		X	X		X		X	X	X	X		X	X*	X*			X*	
Types of governance in mental health																							
Responsible	X	X*	X	X	X*		X	X		X	X*	X	X	X	X	X*	X*	X*	X*		X*	X	X*
Accountability processes	X		X	X				X		X		X		X*	X*							X*	
Participatory				X	X*					X				X*	X*					X*			X*
Collaborative			X	X						X													X*
Based on evidence	X		X	X				X		X		X			X	X*	X*	X			X*		
Communicative				X										X*	X*		X*						
Equitable																X*						X*	
Regulatory																			X*			X*	X*

X* Means that the standard is still not available.

The governance in mental health policies was delimited service delivery and organization. The related studies in service delivery strategy and planning^2^ ^,^ ^8^ ^,^ ^10^ ^,^ ^13^ ^-^ ^15^ ^,^ ^21^ ^,^ ^27^ ^,^ ^34^ ^,^ ^35^ ^,^ ^37^ ^,^ ^40^ ^,^ ^46^ ^-^ ^48^ refered to the decision-making processes, with focus beyond the clinical setting. We referred to the social, communal, juridical and legal context. The studies with a perspective on the organization and the quality of the services^2^ ^,^ ^8^ ^,^ ^10^ ^,^ ^12^ ^-^ ^14^ ^,^ ^26^ ^,^ ^29^ ^,^ ^32^ ^,^ ^34^ ^,^ ^35^ ^,^ ^39^ ^,^ ^40^ ^,^ ^43^ had an exclusively clinical approach (Table 3).

### Actors and Social Regulations

Most studies described strategic actors. Less than half explored the relevant actors^10^ ^,^ ^12^ ^,^ ^13^ ^,^ ^26^ ^,^ ^34^ ^,^ ^35^ ^,^ ^39^ ^,^ ^40^ ^,^ ^46^ ^,^ ^47^. Two studies focused on the perspective of users on decision-making^10,12.^. One of them^13^ reinforced that the point of view of relevant actors in developing a mental health policy be included, such as scholars, religious leaders, traditional healers, governmental and non-governmental civil organizations, as well as actors from sectors other than health, such as education, justice and social development, in order to know the particular need for care in mental health^13^. The actors are part of multidisciplinary working groups^8^, formed by managers, directors, clinicians, lawmakers and users, with different levels of power and control within the system. They develop norms that ensure the involvement and responsibility of both providers and users^26^.

The leaders have a clear understanding of their responsibility for deciding on a policy and implementing it; they adhere to the process as required by the regulations; they have appropriate coordination at various levels of application and monitoring mechanisms^13^. In this process, the users join the decision-making process, with focus on the client, direct care of the clinical work team^10^ ^,^ ^42^ and under an ethical framework^29^, thus changing the traditional view of mental health care^47^. The studies highlight that the level of commitment depends on age, educational level, severity of mental condition, ethnicities, cultural differences (value and belief systems) and exact circumstances. We acknowledge the importance of enabling users with mental disorders to increase their participation in decision-making^10^; provide them with further guidance contribute to their empowerment^21^, even when the users participate in clinical trials^6^.

We identified several social regulations. The actors refer to meta-norms to endorse the decision-making^10^ ^,^ ^12^ ^,^ ^13^ ^,^ ^26^ ^,^ ^27^ ^,^ ^34^ ^,^ ^35^ ^,^ ^39^ ^,^ ^40^ ^,^ ^46^; others adopt as a benchmark the constituent norms^10^ ^,^ ^12^ ^,^ ^13^ ^,^ ^26^ ^,^ ^27^ ^,^ ^35^ ^,^ ^39^ ^,^ ^40^; the regulatory norms^10^ ^,^ ^13^ ^-^ ^15^ ^,^ ^21^ ^,^ ^26^ ^,^ ^29^ ^,^ ^43^; and a small number describe the importance of informal norms^10^ ^,^ ^12^ ^,^ ^21^ ^,^ ^26^.

### The Governance Process and Decision Spaces

Studies noted necessary measures in developing mental health policies: exhaustively disseminating the policy; including communication among various levels of the health system; relying on a very clear articulation of the objectives, functions and responsibilities in order to ensure the successful implementation of the policy in the long term^13^, and strengthen the leadership and implement it by trained personnel throughout the country^3^ ^,^ ^43^. Otherwise, there exist differences in resources allocated to service delivery in the country’s interior. This reinforces inequality^27^ and a range of problems related to: conflicts within the system; different organizational culture; lack of responsibility, trust, common vision and shared goals; asymmetry of power, inflexibility; and regulatory mechanisms within the organization^21^.

The governance as a process must include accountability, oversight, controls, systematic reviews of processes and practices in healthcare. The process must ensure that the entire participating team is aware of the results of its collective actions, performance indicators and assessment tools^2^ ^,^ ^15^. It is necessary to adopt a set of principles in the interaction process in decision-making processes, in order to bridge the gap between the management culture and the individual health care^39^; reduce bureaucracy and facilitate research^29^.

The collaboration in service delivery is a basic component of care systems since their creation, as to roles, responsibilities and accountability^21^.

The analysis focused on the participation at a national and governmental level to address the needs in mental health. Challenges arise within the health system itself, such as facing stigma in decision-making, thus underscoring the participation of more government sectors to achieve favorable outcomes^13^.

### Nodal Points

The review of the literature allowed to identify problems related to the governance: scarce research resources^48^, particularly in lower and middle-income countries; functional fragmentation of the mental healthcare systems in these countries; lack of a national health system; lack of policies directly affect the organization and the service delivery^40^; additionally, policies that are improperly conducted^46^. This problem arises, from the perspective of the key actores, from: government budget cuts; financing methods at various operation levels of the health system; forms of payment and recovery; outside community care; psychiatric hospital reforms; among others^40^. While there has been important progress in mental health systems, the actors face problems that could not have been reverted into the structure and functioning of mental health services^8^ ^,^ ^10^ ^,^ ^12^ ^,^ ^26^ ^,^ ^39^ ^,^ ^40^.

## DISCUSSION

In the papers reviewed^8^ ^,^ ^10^ ^,^ ^12^ ^,^ ^29^ ^,^ ^32^ ^,^ ^34^, the concept of governance refers to a process of decision-making that reflects values, centered attention to the patient and based on evidence, where all the actors in the health system intervene: from the providers to the users, with well defined roles, responsibility and accountability. The governance is key in the making of policies and programs of mental health, because it determines structure, service provision and results in mental health. It is a concept closer to the norm and restricted only to the clinical area.

Little attention has been given to the concept of governance in mental health. Despite the fact that countries such as England, Australia and New Zealand have somehow adopted the system, when incorporating the patient in the make of decisions^8^ and the maintenance of the patterns of quality, this approach was not adopted in other countries, i.e., the United States. The main problem in that country seems to be its on health system that has a different structure of organization, where the services are provided segmented. Also, not all the providers agree on incorporating the users in the process of taking decisions. Providers argue that they have the legal responsibility and mention the lack of resources, infrastructure and the medical condition of the own patients as barriers for adopting the system^10^ ^,^ ^12^ ^,^ ^14^.

Sharing decision is still on debate, a discussion reinforced by the tradition of the providers of taking all the decisions related to what is best for the users, relied solely on their medical knowledge^12^. However, users must acquire abilities and training in order to participate in the decision making process and this fact may favor their satisfaction and empowerment^8^ ^,^ ^10^ ^,^ ^12^ ^,^ ^26^ ^,^ ^40^. The possibility of participation depends on the level of illness of the patient, their access to economical, technological and information resources. This fact applies not only to the clinical field but also to their personal lives. The empowerment of the users directly contributes to their participation and recovery of their health^1^.

The different actors in the mental health system organize a way of solving the main problems that derive from the necessity of attention in mental health throughout many strategies. The activities involve government actors, civil organizations, community leaders, researchers and other social leaders, networks of support and non-profit organizations all together to come to solutions and apply them. That means that the solution of mental health problems, in particular, is a task not only of the government, but can also use the help of other actors in different social sectors.

For example, a policy of global impact such as the deinstitutionalization of the patients, in which there is a decrease in beds of psychiatric hospitals, implies that every health system, in every country, supports the new structure and organization of the health system, and the results will always be different. In the case of the United States, measures were taken focused on the health system (decrease of government budget destined to psychiatric hospitals and increase of the private participation). The strategies were not followed by a wholesome policy, such as providing services of mental attention in the communities. This fact created another problem: the raise in demanding of attention in the service of hospital emergencies and abandon of the treatment^14^. In the case of the American and Australian health systems, the strategies were followed by the participation of other social sectors in order to make sure the continuance of the treatments. Nonetheless, their systems have faced other problems when implemented, such as the fact of lack of specialized personnel in other spheres that the clinical, and the difficult articulation between primary and specialized attention^8^ ^,^ ^15^.

Policies of deinstitutionalization and decrease of the number of beds in psychiatric hospitals have also been adopted in Latin American countries. In Chile, strategies akin to England’s have been adopted for securing the continuity of the patients’ treatment, also creating protected environments, and psychiatric beds in General Hospitals for emergencies. Chronic patients have faced challenges in the process of rehabilitation and social integration, because many patients had lacking social networks and a severe intelectual and physical deterioration. Other countries such as Brazil, Argentina and Mexico, besides facing the same problems as Chile, deal with another one which directly concerns the health system: the fragmentation of services and lack of budgetary resources. Like the United States, in these countries both problems represent barriers for a good development of mental health policies.^10^

The clinic governance, key framework of this paper focused on mental health, has had good results, showing that it can provide a good model to be followed inside the structure and the development of policies in mental health ^2^ ^,^ ^15^ ^,^ ^32^ ^,^ ^35^ ^,^ ^37^ ^,^ ^39^. The experiences show that arrangement and organization are pivotal in the health policies and the same principles can be applied when it comes to the care of people with mental diseases and to think about successful experiences of implementing new public policies on mental health.

An analytical approach on governance allows to understand how to carry out the process, the characteristics of the main actors, the norms which rule its function and to identify the problems and the factors involved in the politic, social and economical context that characterize a mental health system.

**Figure 2:**
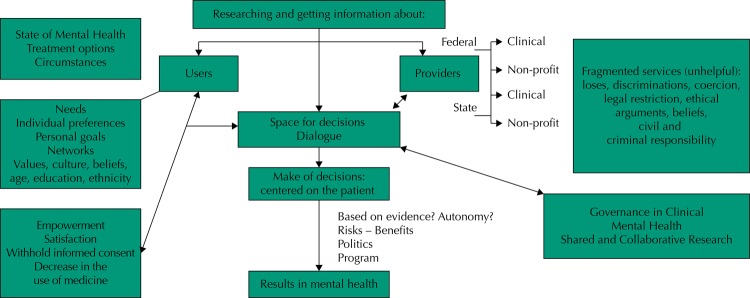
Governance scheme after the systematic revision.

In order to summarize and based on the levels of analysis proposed in this study, we were able to build a scheme of the process of governance (Figure 2). We propose the following concept of governance in mental health:

a process of decision-making whereas all the actors in the health system intervene, from providers to users, all with well defined roles satisfying their needs of attention in mental health, with the attention centered on the patient and placed in evidence, responsibility and accountability tool.

This concept, despite the fact that aims to evolve all the more relevant studies in the area, still is perfectible, in function of the context behind the mental health system.

The health systems in Latin America, like those in Mexico, Brazil and Chile, in general, are rooted in the principals of equity and responsibility before the law and most countries in the continent recognize the right to health in their constitutions. That said, being the governance a process of decision-making, it can be argued that this process roots its principles in the same of the general health system, as it occurs in countries like England. However, it is a subject that still requires more study.

The approaches of governance in mental health that countries such as England and New Zealand already have, where the health system is national, identify that governance is a responsible process, based on evidence and which includes all the actors in the system. For that reason, the making of effective health policies compels the sharing of more actors in society. This is a desirable model, but hard to follow in some health systems in Latin America due to its structure and organization. Chile’s health system has resemblances with it; however, the country faces the problem of low budget to full implement it. Despite United States health system’s structure and organization is unique in many ways, it shares some coincidences with countries such as Brazil and Mexico, especially when it comes to fragmentation and the lack of articulation in the provision of services of mental health, besides the low budget for a efficient implementation of existents public policies. This is a subject that still deserves attention in forthcoming researches.

## Footnotes

a United Nations Governance for sustainable human development: UNDP policy document. New York: United Nations Development Programme; 1997.

b Comission of the European Communities. Report from the Comission on European Governance. Luxembourg: Office for Oficial Publications of the European Communities; 2003. Available from: http://ec.europa.eu/governance/docs/comm rapport en.pdf

c World Health Organization. Everybody’s business: strengthening health systems to improve health outcomes: WHO’s framework for action. Geneva: 2007.

d World Health Organization. Department of Health System Good governance for health. Geneva: 1988.

e Arredondo A, Orozco E, Wallace S, Rodríguez M. Gobernanza en sistemas de salud: conceptos, aportes y evidencias para el avance de las estrategias de protección social en la salud de los migrantes. Cuernavaca (Mex): Instituto Nacional de Salud Pública; 2010.

f Shukla M, Johnson LK. Governing for health in low- and middle- income countries: perspectives from the field. Prepared by USAID Leadership Management and Government Project. Arlington, VA: Management Sciences for Health; 2012.

g Urrutia G, Bonfill X. Declaración PRISMA: una propuesta para mejorar la publicación de revisiones sistemáticas y Metaanálisis. *Med Clin* (Barc) 2010;135:507-11.

## References

[1] Arredondo A, Orozco E, Aviles R (2015). Evidence on equity, governance and financing after health care reform in Mexico: lessons for Latin American countries. Saude Soc.

[2] Arya D, Callaly T (2005). Using continuous quality improvement to implement a clinical governance framework in a mental health service. Australas Psychiatry.

[3] Azami-Aghdash S, Tabrizi JA, Sadeghi-Barzargani H, Hajebrahimi S, Naghavi-Behzad M (2015). Developing performance indicators for clinical governance in dimensions of risk management and clinical effectiveness. Int J Qual Health Care.

[4] Barbazza E, Tello JE (2014). A review of health governance: definitions, dimensions and tools to govern. Health Policy.

[5] Bazzani R (2010). Gobernanza y salud: aportes para la innovación en sistemas de salud. Rev Salud Publica.

[6] Berge E, Ford GA, Bath PMW, Stapf C, Worp HB, Demotes J (2015). Regulation and governance of multinational drug trials in stroke: barriers and possibilities. Int J Stroke.

[7] Brinkerhoff DW, Bossert T (2008). Health governance: concepts, experiences, and programming options.

[8] Callaly T, Arya D, Minas H (2005). Quality, risk management and governance in mental health: an overview. Australas Psychiatry.

[9] Carlson V, Chilton MJ, Corso LC, Beitsch LM (2015). Defining the functions of public health governance. Am J Public Health.

[10] Curtis LC, Wells SM, Pennet DJ, Ghose SS, Mistler LA, Mahone IH (2010). Pushing the envelope: shared decision making in mental health. Psychiatr Rehabil J.

[11] Dlouhy M (2014). Mental health policy in Eastern Europe: a comparative analysis of seven mental health systems. BMC Health Serv Res.

[12] Drake RE, Deegan PE, Rapp C (2010). The promise of shared decision making in mental health. Psychiatr Rehabil J.

[13] Draper CE, Lund C, Kleintjes S, Funk M, Omar M, Flisher AJ (2009). Mental health policy in South Africa: development process and content. Health Policy Plann.

[14] Frueh BC, Grubaugh AL, Lo Sasso AT, Jones WJ, Oldham JM, Lindrooth RC (2012). Key stakeholder perceptions regarding acute care psychiatry in distressed publicly funded mental health care markets. Bull Menninger Clin.

[15] Gask L, Rogers A, Campbell S, Sheaff R (2008). Beyond the limits of clinical governance? The case of mental health in English primary care. BMC Health Serv Res.

[16] Gostin LO (2014). Healthy living needs global governance. Nature.

[17] Goodman C, Davies SL, Gordon AL, Meyer J, Dening T, Gladman JR (2015). Relationships, expertise, incentives, and governance: supporting care home residents’ access to health care: an interview study from England. J Am Med Dir Assoc.

[18] Greer SL, Lillvis DF (2014). Beyond leadership: political strategies for coordination in health policies. Health Policy.

[19] Hastings SE, Armitage GD, Mallinson S, Jackson K, Suter E (2014). Exploring the relationship between governance mechanisms in healthcare and health workforce outcomes: a systematic review. BMC Health Serv Res.

[20] Helgesen M (2014). Governance of public health: Norway in a Nordic context. Scand J Public Health.

[21] Hodges S, Ferreira K, Mowery D, Novicki E (2013). Who’s in charge here? Structures for collaborative governance in children’s mental health. Adm Soc Work.

[22] Hufty M (2010). Gobernanza en salud pública: hacía un marco analítico. Rev Salud Publica.

[23] Hufty M, Báscolo E, Bazzani R (2006). Gobernanza en salud: un aporte conceptual y analítico para la investigación. Cad Saude Publica.

[24] Kaufmann D, Kraay A, Zoido-Lobatón P (1999). Governance matters.

[25] Knight KM, Kenny A, Endacott R (2015). Gaps in governance: protective mechanisms used by nurse leaders when policy and practice are misaligned. BMC Health Serv Res.

[26] Linhorst DM, Eckert A (2003). Conditions for empowering people with severe mental illness. Soc Serv Rev.

[27] Lund C, Kleintjes S, Kakuma R, Flisher AJ (2010). Public sector mental health systems in South Africa: inter-provincial comparisons and policy implications. Soc Psychiatry Psychiatr Epidemiol.

[28] Marais DL, Petersen I (2015). Health system governance to support integrated mental health care in South Africa: challenges and opportunities. Int J Ment Health Syst.

[29] Meenaghan A, O´Herlihy A, Durand MA, Farr H, Tulloch S, Lelliott P (2007). A 55 kg paper mountain: the impact of new research governance and ethics processes on mental health services research in England. J Ment Health.

[30] Meier B, Ayala AS (2014). The Pan American Health Organization and the mainstreaming of human rights in regional health governance. J Law Med Ethics.

[31] Minas H (2009). International observatory on mental health systems: structure and operation. Int J Ment Health Syst.

[32] Newman DW, Kellett S, Beail N (2003). From research and development to practice-based evidence: clinical governance initiatives in a service for adults with mild intellectual disability and mental health needs. J Intell Disabil Res.

[33] Nicholson C, Jackson CL, Marley JE (2014). Best-practice integrated health care governance: applying evidence to Australia’s health reform agenda. Med J Aust.

[34] Niemi M, Thanh HT, Tuan T, Falkenberg T (2010). Mental health priorities in Vietnam: a mixed-methods analysis. BMC Health Serv Res.

[35] O’Connor N, Paton M (2008). ‘Governance of’ and ‘Governance by’: implementing a clinical governance framework in an area mental health service. Australas Psychiatry.

[36] Prats i Català J (2001). Gobernabilidad democrática para el desarrollo humano: marco conceptual y analítico. Inst Desarro.

[37] Rogers A, Campbell S, Gask L, Sheaff R, Marshall M, Halliwell S (2002). Some National Service Frameworks are more equal than others: implementing clinical governance for mental health in primary care groups and trusts. J Ment Health.

[38] Semrau M, Evans-Lacko S, Alem A, Ayuso-Mateos JL, Chisholm D, Gureje O (2015). Strengthening mental health systems in low- and middle-income countries: the Emerald programme. BMC Med.

[39] Singh B (2003). Why clinical governance is important: an approach to the resolution of managerial and professional conflict in mental health. Australas Psychiatry.

[40] Stelk W, Slaton E (2010). The role of infrastructure in the transformation of child-adolescent mental health systems. Adm Policy Ment Health.

[41] Tambor M, Pavlova M, Golinowska S, Sowada C, Groot W (2013). The formal-informal patient payment mix in European countries. Governance, economics, culture or all of these?. Health Policy.

[42] Torres A, Kunishige N, Morimoto D, Hanzawa T, Ebesu M, Fernández J (2015). Shared governance: a way to improve the care in an inpatient rehabilitation facility. Rehabil Nurs.

[43] Van Houtte E (2010). Validating certification in a recovery focused mental health system. Psychiatr Rehabil J.

[44] Wahlbeck K (2015). Public mental health: the time is ripe for translation of evidence into practice. World Psychiatry.

[45] Wiktorowicz ME, Fleury MJ, Adair CE, Lesage A, Goldner E, Peters S (2010). Mental health network governance: comparative analysis across Canadian regions. Int J Integr Care.

[46] Wolff N (2002). Risk, response, and mental health policy: learning from the experience of the United Kingdom. J Health Polit Policy Law.

[47] Woltmann EM, Whitley R (2010). Shared decision making in public mental health care: perspectives from consumers living with severe mental illness. Psychiatr Rehabil J.

[48] Yasamy MT, Maulik PK, Tomlinson M, Lund C, Van Ommeren M, Saxena S (2011). Responsible governance for mental health research in low resource countries. PLoS Med.

